# Pharmacokinetic/Pharmacodynamic Modeling of a Cell-Penetrating Peptide Phosphorodiamidate Morpholino Oligomer in *mdx* Mice

**DOI:** 10.1007/s11095-021-03118-5

**Published:** 2021-10-20

**Authors:** Marie Claire Mukashyaka, Chia-Ling Wu, Kristin Ha, Jianbo Zhang, Jenna Wood, Samantha Foley, Bryan Mastis, Nino Jungels, Huadong Sun, Mohammad Shadid, Shawn Harriman, John R. Hadcock

**Affiliations:** 1 Translational Sciences Group, Sarepta Therapeutics, Inc., 215 First St., Cambridge, MA 02142 USA; 2 Biology Group, Sarepta Therapeutics, Inc., Cambridge, MA USA; 3 Clinical Pharmacology Group, Sarepta Therapeutics, Inc., Cambridge, MA USA

**Keywords:** Duchenne muscular dystrophy, Peptide-conjugated phosphorodiamidate morpholino oligomers, Phosphorodiamidate morpholino oligomers, Pharmacokinetic/pharmacodynamic model, Simulation

## Abstract

**Purpose:**

Peptide-conjugated phosphorodiamidate morpholino oligomers (PPMOs) have shown promise in treating Duchenne muscular dystrophy (DMD). We evaluated a semi-mechanistic pharmacokinetic (PK) and pharmacodynamic (PD) model to capture the relationship between plasma and muscle tissue exposure/response in *mdx* mice treated by mouse surrogate PPMO.

**Methods:**

A single or repeated (every 4 weeks for 20 weeks) intravenous PPMO dose was administered to *mdx* mice (n = 6/timepoint). A PK/PD model was built to characterize data via sequential modeling. A 2-compartment model was used to describe plasma PK. A simultaneous tissue PK/PD model was subsequently developed: 2-compartment model to describe muscle PK; linked to an indirect response model describing stimulation of synthesis of skipped transcript, which was in turn linked to stimulation of synthesis of dystrophin protein expression.

**Results:**

Model performance assessment via goodness-of-fit plots, visual predictive checks, and accurate parameter estimation indicated robust fits of plasma PK and muscle PK/PD data. The model estimated a PPMO tissue half-life of 5 days—a useful parameter in determining the longevity of PPMOs in tissue and their limited accumulation after multiple doses. Additionally, the model successfully described dystrophin expression after single dosing and associated protein accumulation after multiple dosing (increasing ~ twofold accumulation from the first to last dose).

**Conclusions:**

This first PK/PD model of a PPMO in a DMD disease model will help characterize and predict the time course of PK/PD biomarkers in *mdx* mice. Furthermore, the model framework can be used to develop clinical PK/PD models and can be extended to other exon-skipping therapies and species.

**Supplementary Information:**

The online version contains supplementary material available at 10.1007/s11095-021-03118-5.

## Introduction

Duchenne muscular dystrophy (DMD) is a rare, X-linked, fatal, degenerative neuromuscular disease affecting ~ 1 in every 3500 to 5000 males born worldwide [1–4]. DMD is caused by mutations in the *DMD* gene, resulting in little or no production of full-length dystrophin [1, 5–7]. Dystrophin, a 427 kDa cytoskeletal protein required for muscle fiber stability, links the sarcomere and the extracellular matrix. In the absence of dystrophin, the dystrophin-associated protein complex is destabilized, resulting in repeated muscle degeneration and regeneration, and replacement of muscle fibers by fat and fibrosis. DMD manifests in patients as progressive muscle weakness, with many patients losing ambulation by age 8 to 14, and generally leads to life-threatening complications including respiratory and cardiac failure [8, 9].

Antisense oligonucleotide (ASO) therapy has been approved by FDA for treatment of some populations of DMD, and is rationally designed to induce skipping of specific exons at the pre-mRNA level to restore the reading frame, producing truncated—yet functional—dystrophin [5, 7, 10–12]. Phosphorodiamidate morpholino oligomers (PMOs), a class of exon skipping therapies, are an established treatment for patients with DMD; peptide-conjugated PMOs (PPMOs) enhance cell permeability and have also shown promise in treating these patients [1, 13–17]. The time-courses of pharmacokinetics (PK) and pharmacodynamics (PD) have been characterized for other ASO classes, such as 2ʹ-*O*-methyl phosphorothioates targeting exon skipping [18, 19], 2ʹ-*O*-(2-methoxyethyl) modified ASOs targeting mRNA gene knockdown [20–22], and siRNA targeting aminolevulinate synthase 1 mRNA [23]. Here, we report the first semi-mechanistic PK/PD model for PPMO in *mdx* mice using RC-1001, a mouse-surrogate PPMO. RC-1001 induces skipping of *DMD* exon 23 containing a point mutation, resulting in a stop codon in the *DMD* gene, restoring the reading frame in *mdx* mice.

RC-1001 was used to develop a plasma PK model to describe plasma concentration over time, and was also used to develop an integrated plasma-tissue PK/PD model to predict tissue concentration, dystrophin production and its intermediate biomarker, skipped transcript. In patients with DMD, muscle biopsy for analysis of treatment response is complicated and not feasible for longitudinal sampling. A PK/PD model describing time-course of tissue exposure and response would be helpful in understanding and predicting the treatment response in patients over time. Additionally, the current model—when scaled to clinical settings—can be used in simulations to inform the design of clinical dosage paradigms of PPMO molecules.

## Materials and Methods

### Chemicals

RC-1001, a mouse-specific surrogate PPMO, was manufactured at Sarepta Therapeutics, Inc. by conjugating a proprietary cell-penetrating peptide to M23D (+7–18) PMO sequence. All RC-1001 doses were formulated in saline before administration.

### Animals

*mdx* male mice (C57BL/10ScSn-*DMD*^*mdx*^/J, stock #001801; The Jackson Laboratory, n = 3 per timepoint for saline-treated control and n = 6 per timepoint for PPMO-treated groups) and male wild-type (WT) mice (C57BL/6J, The Jackson Laboratory, n = 3 per timepoint) were housed at the Sarepta animal facility and given food (Labdiet 5P76; ScottPharma Solutions) and water ad libitum. After 3 days’ acclimation, 6- to 8-week-old mice were randomized into treatment groups. All procedures were approved by and conducted under guidance from Sarepta’s Institutional Animal Care and Use Committee.

### Animal Experiments

#### Single Dose to Measure Plasma PK

RC-1001 at doses of 10, 40, and 80 mg/kg was administered to WT mice (n = 3 per dose per timepoint) via tail vein intravenous (IV) bolus injection. Blood samples of 0.5 mL were collected in K_2_EDTA tubes containing 10 µL of 200 mM 4-(2-Aminoethyl) benzenesulfonyl fluoride hydrochloride (AEBSF) in total volume of 500 µL, with a final concentration of 4 mM AEBSF. Blood samples were collected from three animals at 5 and 30 min and 2, 4, 8, 12, and 24 h post-injection via cardiac puncture. Each animal contributed to one datapoint. Samples were mixed gently and centrifuged (10 min, 4 °C, 1400×*g*). Resulting plasma was separated and stored at − 80 °C until analysis.

#### Single Dose for Longitudinal PK/PD

RC-1001 was administered to *mdx* mice at doses of 0, 40, and 80 mg/kg (n = 6 and n = 3 per dose per timepoint in RC-1001- and control-treated groups, respectively). Age- and sex-matched WT control mice received vehicle control via IV bolus (n = 3 per timepoint). Mice were euthanized at 1, 2, 5, 7, 10, 14, and 28 days post-injection by carbon dioxide inhalation/anesthesia, followed by exsanguination. Each animal contributed to one corresponding PK and PD datapoint. Tissue concentration was measured in quadriceps, and exon skipping and dystrophin were measured in biceps. Tissue samples (quadriceps and biceps) were collected, flash-frozen in liquid nitrogen, and stored at − 80 °C until analysis.

#### Repeated Dose for Longitudinal PK/PD

*mdx* mice received repeated IV bolus injections of RC-1001 at time zero, then at 4-week intervals for a total of 20 weeks at 0, 40, and 80 mg/kg (n = 3 and n = 6 per dose per timepoint in saline control- and RC-1001-treated groups, respectively). Age- and sex-matched WT control mice received once every 4-weeks repeated bolus IV of saline control treatment (n = 3 per timepoint). Mice that received 1, 2, 3, 4, 5, and 6 treatments were euthanized 28, 56, 84, 112, 140, and 168 days, respectively, after the first injection. Each animal contributed to one corresponding PK and PD datapoint. Quadriceps and biceps were dissected, flash-frozen in liquid nitrogen, and stored at − 80 °C until analysis. Tissue concentration was measured in quadriceps after first and last dose (days 28 and 168); exon skipping and dystrophin were measured in biceps at each time point.

### Measuring Plasma and Tissue Concentrations of PPMO

A liquid chromatography–tandem mass spectrometry method was developed to determine RC-1001 in mouse K_2_EDTA plasma treated with 4 mM AEBSF; NG-12-0064 was the internal standard. RC-1001 and the internal standard were extracted by solid-phase extraction and filtration from mouse plasma. Reversed-phase high-pressure liquid chromatography separation was achieved with a Waters XBridge column and sample analysis in TIS positive mode of SCIEX API 5000. The calibration curve range was from 10 to 2000 ng/mL with low, mid and high quality controls included. The lower limit of quantification was 10 ng/mL, with the coefficient of variation (%CV) less than 15%.

RC-1001 tissue samples were collected and homogenized in 50 mM Tris–HCl pH 7.5 buffer. Homogenates were then subjected to proteinase-K/Trypsin digestion to convert all potential metabolites to one end product of PMO-Gly before solid-phase extraction. Digested homogenates and internal standard were loaded and washed with ammonium acetate buffer, then eluted with H_2_O/ACN/FA (70/30/5) twice in Waters HLB SPE 96-well plates. The eluate was concentrated under nitrogen gas, then injected to ultra-performance liquid chromatography–high-resolution mass spectrometry, followed by parallel reaction monitoring mass spectrometry (Thermo Fisher Q Exactive Plus Hybrid Quadrupole-Orbitrap) quantitation. Lower limit of quantification of all tissues was 60–100 ng/g based on the type of tissue, with the %CV less than 25%.

### Measuring Exon Skipping Levels in Muscle Tissues

Tissue samples were homogenized using Matrix S by a FastPrep-24 5G instrument (MP Biomedicals). Homogenates were centrifuged (10 min, 4 °C, 12,000×*g*). Resultant supernatant lysates were loaded onto a 96-well Illustra RNAspin 96 RNA isolation column (GE Healthcare Life Sciences); total RNA was isolated per the manufacturer’s instructions. After eluting with 50 μL of RNase-free water, total RNA concentration from each sample was measured using a NanoDrop 2000 spectrophotometer (Thermo Fisher Scientific). 250 ng of total RNA were reverse transcribed for cDNA using SuperScript IV First-Strand Synthesis Kit (Invitrogen, Cat#18091200) with random hexamers. The cDNA samples were diluted 1:5 with DNase- and RNase-free water; 3 µL diluted cDNA was added into each digital droplet polymerase chain reaction (ddPCR) using ddPCR Supermix for Probe (Bio-Rad Laboratories, Inc.) with these primer probe sequences: FW-GGATCCAGCAGTCAGAAAG, RV-ACCAACTAAAAGTCTGCATTG, FAM-AGA CTC GGG AAA TTA CAG AAT CAC (skipped transcript), and HEX-TTG AAG AGA TTG AGG GGC AC (unskipped transcript).

After droplet generation (automated droplet generator, Bio-Rad QX200), PCR cycling was conducted by a C1000 Touch thermocycler (Bio-Rad Laboratories, Inc.) using these parameters: enzyme activation (95 °C, 10 min), denaturation (94 °C, 30 s), annealing/extension (55 °C, 1 min), repeating for denaturation and annealing/extension for 60 cycles, enzyme deactivation (98 °C, 10 min), and holding at 4 °C until data acquisition by a QX200 Droplet Reader (Bio-Rad Laboratories, Inc.). Positive and negative droplet numbers were analyzed by QuantaSoft Software (Bio-Rad Laboratories, Inc.); copy number per microliter of skipped and unskipped transcript levels were quantified.

### Measuring Dystrophin in Muscle Tissues

For protein extraction, frozen tissues were disrupted in homogenization buffer (4 M urea, 125 mM Tris, 4% sodium dodecyl sulfate) with one protease inhibitor cocktail tablet (Roche Applied Science) using a cordless pellet pestle (Kimble Chase Life Science). Protein concentrations were quantified using Pierce BCA Protein Assay (Thermo Fisher Scientific) or *RC DC* Protein Assay (Bio-Rad Laboratories, Inc.) kits, per the manufacturers’ instructions.

For western blot analysis, 50 µg of protein from each lysate or 15 μL of HiMark prestained high-molecular–weight marker (Thermo Fisher Scientific) were loaded onto individual wells of 3% to 8% polyacrylamide Tris–acetate gels (Thermo Fisher Scientific; Bio-Rad Laboratories, Inc.). Antibody dilutions were prepared using anti-dystrophin ab15277, 1:500 (Abcam); anti-alpha actinin (sarcomeric), 1:10,000 (Sigma-Aldrich); horseradish peroxidase (HRP)-conjugated goat anti-rabbit immunoglobulin G (IgG), 1:10,000 (Bio-Rad Laboratories, Inc.); and HRP-conjugated goat anti-mouse IgG, 1:10,000 (Bio-Rad Laboratories, Inc.). Images were captured and band intensities were analyzed using Chemidoc Imaging System and Image Lab software v5.2 (Bio-Rad Laboratories, Inc.).

For dystrophin quantification, pooled protein lysates from WT mice were used as positive controls and lysates from *mdx* mice were used as negative controls. To quantify dystrophin levels, a standard curve with an appropriate range was applied to each gel. The serial diluted points of the standard curve were obtained by mixing the same concentration of the WT and DMD protein lysates. The standard curve was used to determine percentage of WT dystrophin production.

### Noncompartmental Analysis

Noncompartmental analysis (NCA) of the averaged plasma concentration per timepoint was performed in Phoenix WinNonlin (v8.2; Certara USA) using a linear log trapezoidal method. The area under the plasma-time curve (AUC) was calculated with uniform weighting. Elimination half-life (T_1/2_) was calculated by linear regression using logarithmic values of concentration–time data in the terminal phase. Maximum observed concentration (C_max_), plasma clearance (CL), and volume of distribution at the steady state (V_ss_) parameters were also calculated.

### Plasma PK Linearity Analysis

Dose-proportionality of plasma PK was assessed using a power model to define the relationship between PK parameter (y) and dose as follows: $y=\alpha \bullet {dose}^{\beta }$, where *α* and *β* correspond to proportionality constant and exponent. The exponent *β* in the power model was estimated by regressing the natural log-transformed dose. To confirm dose-proportional PK, the estimated power exponent should be close to 1 and a suitable confidence interval (90% CI) should be contained within a prespecified interval (${\beta }_{L},{\beta }_{U}$). This prespecified interval was calculated using an equivalence criterion interval of 0.5–2 suggested by Hummel et al. for exploratory PK linearity assessment as ${(\beta }_{L},{\beta }_{U})=\left(1+\frac{\mathrm{ln}\left({\phi }_{L},\right)}{\mathrm{ln}\left(r\right)},1+\frac{\mathrm{ln}\left({\phi }_{U},\right)}{\mathrm{ln}\left(r\right)}\right)$, where ${\phi }_{L}$ and ${\phi }_{U}$ are lower and higher Hummel’s equivalence criterion of 0.5 and 2, and *r* is the ratio of the highest- to lowest-tested dose levels [24]. Additionally, to assess dose proportionality, plasma PK exposure parameters AUC and C_max_ were normalized to dose level and ANOVA was used to test for a significant difference in dose-normalized PK exposures between dosing groups.

### PK/PD Modeling

**Fig. 1:**
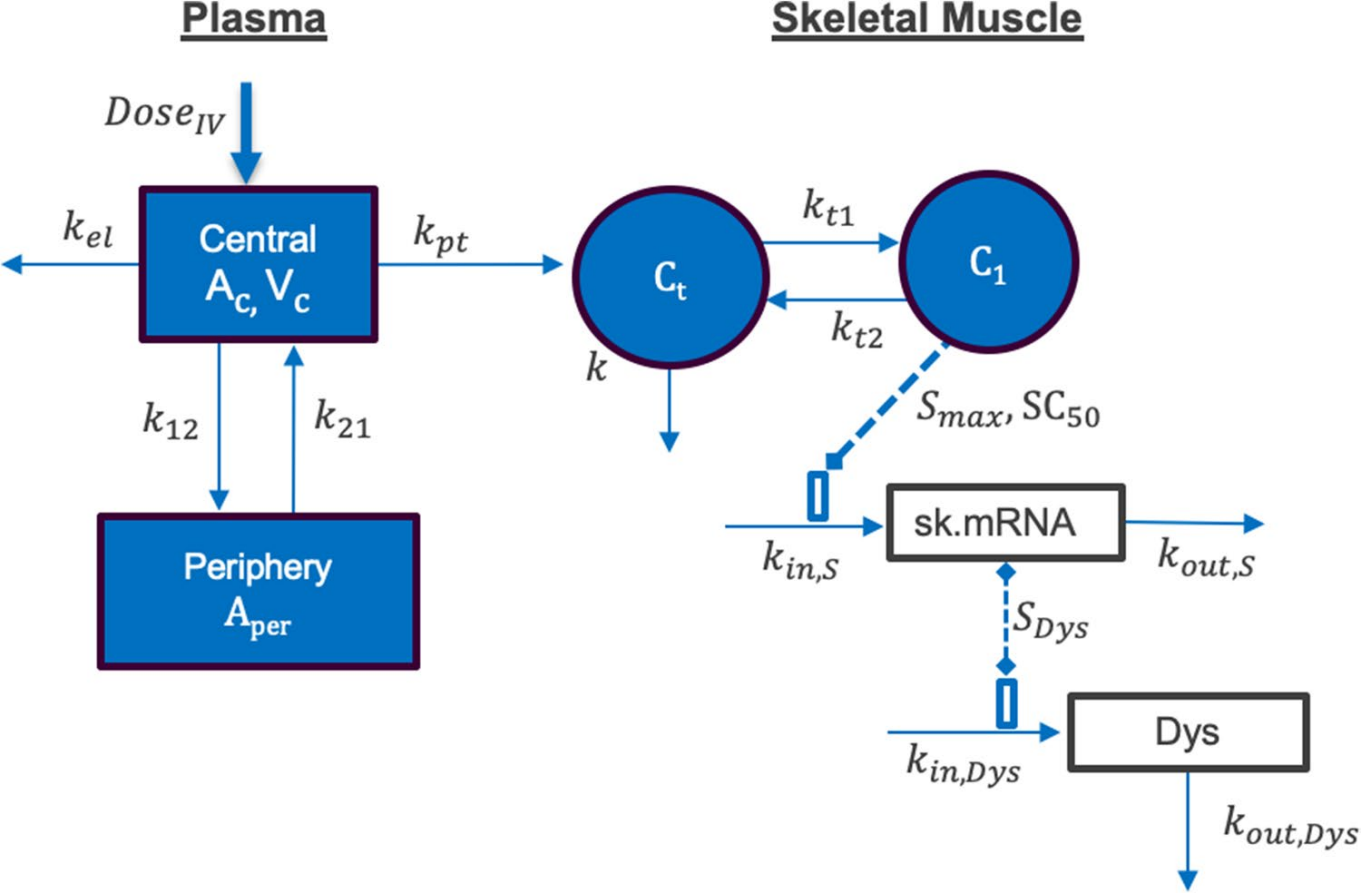
Integrated PK/PD model for exon skipping and dystrophin protein expression by PPMO. Symbols are defined in Table 1

The RC-1001 PK/PD model includes: (1) IV dosing, (2) PPMO plasma disposition (distribution + elimination), (3) uptake/distribution into skeletal muscle tissue, (4) target engagement with pre-mRNA to generate exon skipping, and (5) transcription of the skipped mRNA, producing dystrophin. Figure 1 provides detailed mechanisms built in the model and corresponding parameters. Based on previous work showing similar plasma PPMO exposure in WT and *mdx* mice (unpublished data), the plasma PK model was developed using WT mouse data. A sequential modeling approach was used to link the plasma PK model to the tissue PK/PD model. A plasma PK model was first developed to fit a dataset at 10, 40, and 80 mg/kg single IV bolus dose. A plasma PK model and estimate parameters were used to link plasma PK to tissue PK/PD in a subsequent model. A linear 2-compartment model (Fig. 1) was used to fit the plasma data (Eqs. 1–3).

1 $$\frac{d{A}_{c}}{dt}={k}_{21}\cdot {A}_{per}-\left({k}_{el}+{k}_{12}\right)\cdot {A}_{c} \quad {A}_{c}\left(0\right)=Dose$$

2 $${C}_{p}=\frac{{A}_{c}}{{V}_{c}}$$

3 $$\frac{d{A}_{per}}{dt}={k}_{12}\cdot {A}_{c}-{k}_{21}\cdot {A}_{per}\quad {A}_{per}\left(0\right)=0$$

where A_c_ represents drug amount in the central compartment, A_per_: amount in the peripheral compartment, C_p_: plasma concentration, k_12_ and k_21_: first-order rate constants for distribution from central to peripheral compartments and vice-versa, k_el_: first-order rate constant for elimination, and V_c_: apparent volume of distribution in the central compartment (plasma). Total plasma and intercompartmental clearances (CL and CL_d_) and volume of distribution in peripheral compartment (V_2_) were calculated as derived parameters from model parameter estimates:

$$CL = {k}_{el}\bullet {V}_{c}, C{L}_{d}={k}_{12}\bullet {V}_{c},\ and\ {V}_{2}=\frac{C{L}_{d}}{{k}_{21}}$$

Estimated parameters by the plasma PK model were fixed for subsequent modeling.

A tissue PK/PD model was developed and fit to *mdx* mice tissue PK/PD data from both the single and repeated dose studies. Tissue (skeletal muscle) was modeled as a “biophase” compartment to describe exposure and PD. Plasma concentration is not detectable beyond 24 h, whereas tissue concentration is detectable 28 days after dosing. Hence, tissues may act as a PPMO depot where there is limited redistribution from tissues back to plasma. A similar model was developed for other ASO molecules by Shimizu et al. [21]. Tissue concentration was characterized by a 2-compartment model to describe the biexponential decline in tissue concentration (Eqs. 4–7).

4 $$\frac{d{A}_{t}}{dt}={k}_{pt}\cdot {A}_{c}+{k}_{t2}\cdot {A}_{1}-\left({k}_{t1}+k\right)\cdot {A}_{t}\quad {A}_{t}\left(0\right)=0$$

5 $${C}_{t}=\frac{{A}_{t}}{{V}_{t}}\quad {C}_{t}\left(0\right)=0$$

6 $$\frac{d{A}_{1}}{dt}={k}_{t1}\cdot {A}_{t}-{k}_{t2}\cdot {A}_{1}\quad\quad { A}_{1}\left(0\right)=0$$

7 $${C}_{1}={A}_{1}\bullet \frac{{k}_{t1}}{{k}_{t2}\bullet {V}_{t}}\quad\quad { C}_{1}\left(0\right)=0$$

where A_t_ and A_1_ are the amounts in the tissue central and distribution (peripheral) compartments, C_1_: drug concentration in the tissue peripheral (distribution) compartment, C_t_: drug concentration in the tissue central compartment, k: first-order elimination rate constant in muscle, k_pt_ represents first-order distribution rate constant from the plasma central compartment to the muscle’s central compartment, k_t1_ and k_t2_: first-order tissue inter-compartmental rate constants, and V_t_: volume of distribution in muscle central compartment. PPMO molecules in target tissue organs (skeletal muscle in this model) distribute to the nucleus and engage with pre-mRNA to exert exon skipping. The concentration in the peripheral compartment (C_1_) was assumed as the driver of measured PD effect (exon skipping). Drug concentration in the peripheral tissue compartment was used as the PD driver instead of the central compartment due to observed hysteresis of exon skipping in relation to measured tissue concentration; better performance of the model using the peripheral compartment as the PD driver; and suitability of the PPMO’s mechanism of action, where the drug distributes to a tissue sub-compartment (nucleus) and complementarily binds with pre-mRNA to cause exon skipping.

The measured tissue concentration in quadriceps muscle was assumed to be the concentration in the central compartment of the skeletal muscle model. Because biceps muscle tissue from a single mouse could not yield sufficient homogenate to assess all biomarkers, tissue concentration was measured in the quadriceps, whereas PD biomarkers (exon skipping and dystrophin) were measured in the biceps. Based on previous studies showing comparable tissue concentrations in skeletal muscles (quadriceps and biceps; unpublished data), quadriceps tissue concentration was used in the current model and linked to PD effect (exon skipping and dystrophin) in the biceps muscle after single- and multiple-dose studies.

For the PD model, skipped mRNA message was modeled with a semi-mechanistic indirect response (IDR) model. The mechanism of action of the PPMOs used in this study involves binding to pre-mRNA of a target exon and alternatively splicing out the targeted exon. The resultant mature mRNA with the skipped exon, also referred to as “skipped transcript,” was characterized by an IDR model representing a response resulting from the stimulation of synthesis in the response variable (IDR model 3) [25]. Baseline copy numbers for skipped messages were set to observed values from *mdx* mice receiving vehicle control.

Skipped transcript is transported to the cytoplasm for translation of truncated but functional dystrophin. The skipped transcript was used as the driver of dystrophin synthesis. A linear IDR model 3 was used to describe dystrophin. Because expressed protein in untreated *mdx* mice is below limit of quantification of western blot, the baseline for dystrophin was estimated by the model during early model development, and the estimated value (0.1% WT) was fixed in the final model to reduce the number of estimated parameters. This value was close to %WT (0.07% WT) obtained when the signal in vehicle-treated mice is extrapolated beyond the accurate limit of quantification. Differential equations used to model PD data are presented in Eqs. 8–13.

Skipped transcript:

8 $$\frac{d\left[sk.mRNA\right]}{dt}={k}_{in,s}\bullet \left(1+\frac{{S}_{max}\bullet {C}_{1}^{{\gamma }_{s}}}{S{C}_{50}^{{\gamma }_{s}}+{C}_{1}^{{\gamma }_{s}}}\right)-{k}_{out,s}\bullet [sk.mRNA]$$

9 $$sk.mRNA \left(0\right)=sk.mRN{A}_{0}$$

10 $${k}_{in,s}=sk.mRN{A}_{0}\bullet {k}_{out, s}$$

Dystrophin:

11 $$\frac{d[Dys]}{dt}={k}_{in,Dys}\left(1+{S}_{Dys}\bullet \left[sk.mRNA-sk.mRN{A}_{0}\right]\right)-kou{t}_{Dys}\bullet [Dys]$$

12 $$Dys\left(0\right)=Dy{s}_{0}$$

13 $${k}_{in,Dys}=Dy{s}_{0}\bullet {k}_{out,Dys}$$

where k_in,S_ represents zero-order synthesis rate constant for skipped message, k_out,S_: first-order rate constant for turnover of skipped message, S_max_: maximum drug-induced effect on the production of skipped mRNA, SC_50_: drug concentration that produces 50% of S_max_ in the muscle tissue. ${\gamma }_{s}$ is hill coefficient for skipped message, k_in,Dys_: zero-order rate constant for synthesis of dystrophin, S_Dys_: the linear slope of translation of skipped mRNA to protein, and k_out,Dys_: first-order rate constant for turnover of dystrophin. sk.mRNA, sk.mRNA_0,_ Dys, and Dys_0_ represent change and baseline of number of skipped transcript and dystrophin.

### Data Analysis

A sequential modeling approach was used, in which the plasma PK model was first developed and fixed in the subsequent tissue PK/PD model. The plasma PK model was fit to the single-dose dataset and the tissue PK/PD model was fit to pooled data at each timepoint from both the single- and repeat-dose PK/PD studies. All parameters were estimated using a maximum likelihood estimator (Stochastic Approximation Expectation–Maximization algorithm) in Monolix2020R2 (Lixoft), allowing estimation of fixed effects for each parameter and residual error modeling in one step. Naïve pooled analysis was performed where inter-individual variability was not estimated. Each mouse corresponds with a single datapoint; therefore, it is challenging to estimate individual variability. Data at each dose-level group were treated as coming from a single mouse. Either a proportional model or a combined constant and proportional model was used to describe residual uncertainty in the datasets based on inspection of diagnostic plots. The proportional residual error model assumes that the amount of noise in data is proportional to observed data, whereas the constant error model assumes a constant amount of noise in every observation. The proportional residual error model was used to evaluate plasma and tissue PK data, and dystrophin data. The combined constant and proportional error model was used to evaluate skipped transcript data. An interval data-censoring method built into Monolix was used to handle data below the limit of quantification (BQL). This method, similar to Method 4 in NONMEM, is based on simultaneous modeling of continuous and categorical data, where BQL data are treated as categorical data. The likelihood of BQL observations are maximized with respect to model parameters; and the likelihood of these observations is taken to be the likelihood that these data are indeed BQL [26, 27]. The method censors the BQL data and conditions their likelihood that these observations range from 0 and BQL value (60 ng/g in current analysis). The likelihood of the uncensored data (above BQL data) are conditioned that the observations are greater than 0.

To determine the goodness of fit for the model, predicted vs observed, individual/population fitting, and other diagnostic plots (e.g., plots of residuals) were assessed. Relative standard error (RSE) of each parameter was determined to assess accuracy, where RSE < 50% was considered an accurate estimate. For model validation, visual predictive check (VPC) plots were generated to assess the ability of the model to regenerate data. 1500 simulations were compared with observed data in VPC plots; the 10th, 50th and 90th percentiles of both observed and simulated data were calculated and compared.

GraphPad Prism 8 was used to analyze PK linearity/dose-proportionality by the power model and test of ANOVAs.

## Results

### Noncompartmental Analysis

Plasma exposure parameters were determined for 10, 40, and 80 mg/kg doses by NCA (Table 1). T_1/2_ ranged from 0.32 to 0.74 h, and V_ss_ ranged from 177.4 to 278.9 mL/kg. The systemic CL showed a trending decrease with increasing dose, with average CL of 608.8 mL/h/kg, 587.9 mL/h/kg and 426.8 mL/h/kg at 10, 40, and 80 mg/kg, respectively. This trend is consistent with the slightly more-than-dose–proportional increase in AUC, suggesting that an elimination mechanism is being saturated as dose increases.

**Table 1:** NCA estimated plasma PK parameters, mean (SE), n = 3

Dose (mg/kg)	T_1/2_ (h)	C_max_ (µg/mL)	AUC_inf_ (h*µg/mL)	CL (mL/h/kg)	V_ss_ (mL/kg)
10	0.32 (0.15)	51.3 (9.2)	16.7 (26.0)	608.8 (92.3)	177.4 (52.8)
40	0.53 (0.05)	167.5 (15.1)	68.1 (2.9)	587.9 (24.6)	278.9 (39.3)
80	0.74 (0.05)	399.9 (30.3)	188.2 (15.2)	426.8 (33.0)	248.0 (10.7)

*AUC*_*inf*_ area under the concentration–time curve extrapolated to infinity, *C*_*max*_ maximum observed concentration, *CL* clearance, *n* sample size, *NCA* noncompartmental analysis, *PK* pharmacokinetics, *SE* standard error of mean, *T*_*1/2*_ elimination half-life, *V*_*ss*_ volume of distribution at steady state

### Plasma PK Linearity Analysis

**Fig. 2:**
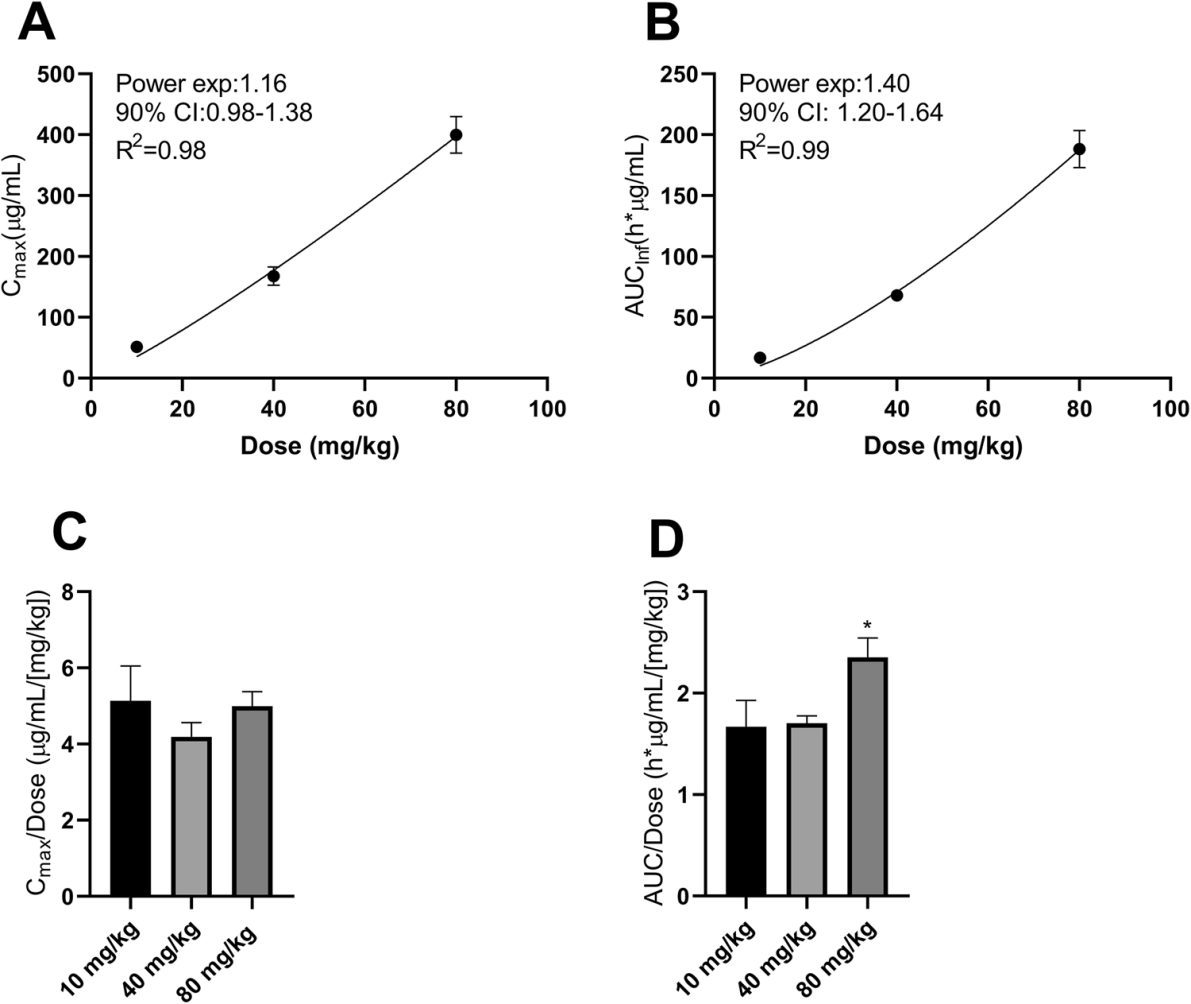
Plasma PK linearity analysis. (**a**, **b**) Plots of plasma exposure versus dose. Line: power model prediction; data points: C_max_ (**a**) and AUC_inf_ (**b**) at shown dose levels. (**c**, **d**) Plots of dose-normalized C_max_ (**c**) and AUC_inf_ (**d**) at tested dose levels. Plots show mean ± SE, n = 3. Statistical significance tested by ANOVA between shown dose levels; *indicates statistical significance (*p*-value < 0.05). *AUC*_*inf*_ area under the concentration–time curve from dosing time extrapolated to infinity, *CI* confidence interval, *C*_*max*_ maximum observed concentration

As the NCA-estimated CL showed a decreasing trend with increasing dose, plasma PK linearity was assessed using a power model and ANOVA. To conclude linear PK, the power model estimated power exponent/slope ($\beta$) value should be close to 1; the estimated 90% CI around $\beta$ should include 1 and be contained within the calculated Hummel’s equivalence criterion interval (HCI). The power model with critical equivalence criterion shows that plasma PK is linear based on C_max_, with $\beta$=1.16, 90% CI 0.98–1.38, HCI 0.67–1.33 (Fig. 2A). On the other hand, the plasma PK is nonlinear based on AUC with $\beta$=1.40, and 90% CI 1.20–1.64 (Fig. 2B).

To further assess plasma PK dose-dependence, an ANOVA test was performed on dose-normalized PK exposure. Plots of dose-normalized C_max_ show dose proportionality, as there is no significant difference between dose-normalized C_max_ at tested doses (10, 40, and 80 mg/kg) (Fig. 2C). By contrast, an ANOVA test on dose-normalized AUC shows a significant difference between 80 mg/kg and other tested dose levels (10 mg/kg, *p* = 0.0108 and 40 mg/kg, *p* = 0.0137), suggesting nonlinearity at 80 mg/kg (Fig. 2D). These results agree with power model analysis showing dose-proportional increases plasma concentration based on C_max_ and greater-than-dose–proportional increases based on AUC.

### Plasma PK Modeling Analysis

**Fig. 3:**
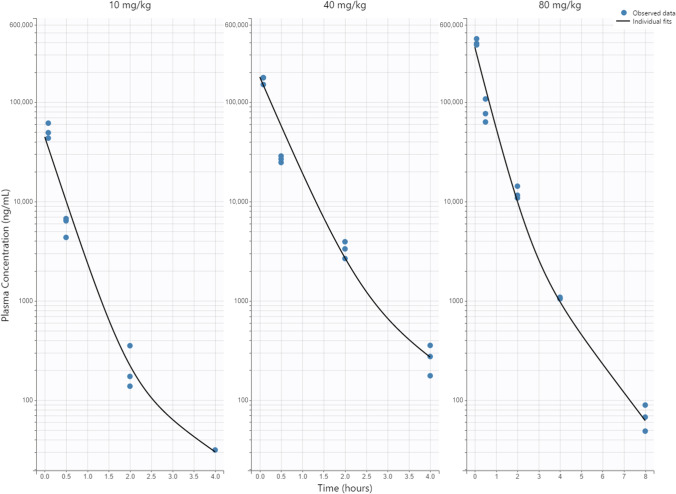
Plasma PK model fitting of PPMO plasma concentration after single IV bolus dose of 10, 40 and 80 mg/kg. Line: model prediction; dots: individual observed plasma concentration (n = 3 per dose per timepoint)

Based on the possibility of nonlinearity from the plasma PK linearity analysis, both linear and nonlinear models were assessed. The nonlinear model showed poor data fitting and parameter estimation compared with the linear model. It is possible that the tested dose range did not fully saturate the nonlinear elimination process. The linear 2-compartment model indicated a robust fit with plasma concentration data and accurately estimated parameters (Fig. 3, Table 2). The high estimated distribution and elimination rate constants correspond to fast distribution and elimination of PPMOs, where plasma concentration is not detectable 24 h post-dose in most animals. This corresponds well with the quick plasma half-life (< 1 h) observed for these molecules. Due to fast dissipation of plasma concentration relative to tissue concentration (plasma undetectable at 24 h, while tissue concentration is detectable up to 28 days post dose), the distribution to tissue PK model was assumed to be a one-way process through parameter k_pt_. The plasma-model estimated parameters were fixed in the subsequent tissue PK/PD model.

**Table 2:** PK/PD model parameter estimates

Model	Parameter	Description	Estimate	RSE %
Plasma PK	${V}_{c}$(mL)	Volume of distribution in central compartment	5.8	62.8
	^a^${k}_{el}$ (1/day)	Elimination rate constant from the central compartment	102.72	23
	^a^${k}_{12}$ (1/day)	First-order rate constant from central to peripheral compartment	21.53	39.1
	^a^${k}_{21}$ (1/day)	First-order rate constant from peripheral to central compartment	30.24	22.4
	^b^CL (mL/h)	Total plasma clearance	24.0	-
	^b^CL_d_ (mL/h)	Intercompartmental clearance	5.2	-
	^b^V_2_ (mL)	Volume of distribution in peripheral compartment	4.1	-
Muscle PK/PD	${k}_{pt}$ (1/day)	First-order rate constant for distribution from central compartment to tissue compartment	0.023	11.7
	${k}_{t1}$ (1/day)	First-order rate constant from tissue central to tissue peripheral compartment	0.37	3.91
	${k}_{t2}$ (1/day)	First-order rate constant from tissue peripheral to tissue central compartment	0.26	3.95
	$k$ (1/day)	First-order elimination rate constant from tissue	0.13	6.79
	V_t_ (mL)	Volume of distribution in muscle central compartment	0.21	16.2
	$S{C}_{50}$ (ng)	Tissue drug concentration required to achieve 50% of S_max_	795.28	10.6
	${S}_{max}$	Maximum stimulation effect of tissue PPMO on skipped transcript	1.41E + 04	16.7
	${k}_{out,S}$ (1/day)	Degradation rate constant for skipped transcript	3.17	3.21
	$sk.mRN{A}_{o}$ (copy#/30 ng RNA)	Skipped transcript copy number at baseline	1.3	Fix
	${\gamma }_{S}$	Hill coefficient for skipped transcript	1.16	1.58
	$Dy{s}_{0}$ (%WT/day)	Dystrophin at baseline	0.10	Fix
	S_Dys_	Linear slope of skipped mRNA translation to dystrophin	0.093	7.79
	${k}_{out,Dys}$ (1/day)	Degradation rate constant for dystrophin	0.044	11.8

*PD* pharmacodynamics, *PK* pharmacokinetics, *PPMO* peptide-conjugated phosphorodiamidate morpholino oligomer, *RSE* relative standard error

^a^Parameter units were converted from 1/h to 1/day to be fixed in the tissue PK/PD model

^b^Parameters calculated as derived parameters from PK model parameter estimates

### Semi-mechanistic Tissue PK/PD Model Development and Validation

No tissue concentration was detected in control-treated mice, hence not used in the analysis. Exon skipping in control-treated mice was substantially lower than in PPMO-treated mice. The average of these values were fixed to baseline skipped copies (sk.mRNA_0_) in the model. Dystrophin expression in vehicle-treated *mdx* mice was below the limit of detection of western blot. Though undetectable by western blot, *mdx* mice express low amounts of dystrophin, hence the baseline value was first estimated and then fixed to the estimated value (0.1%) in the final model. Additionally, when extrapolated, the faint dystrophin signal in vehicle-treated mice corresponded to approximately 0.07% WT, which is close to 0.1. For tissue concentration, a 2-compartment model was used to describe a bi-exponential decline in observed tissue concentration.

**Fig. 4:**
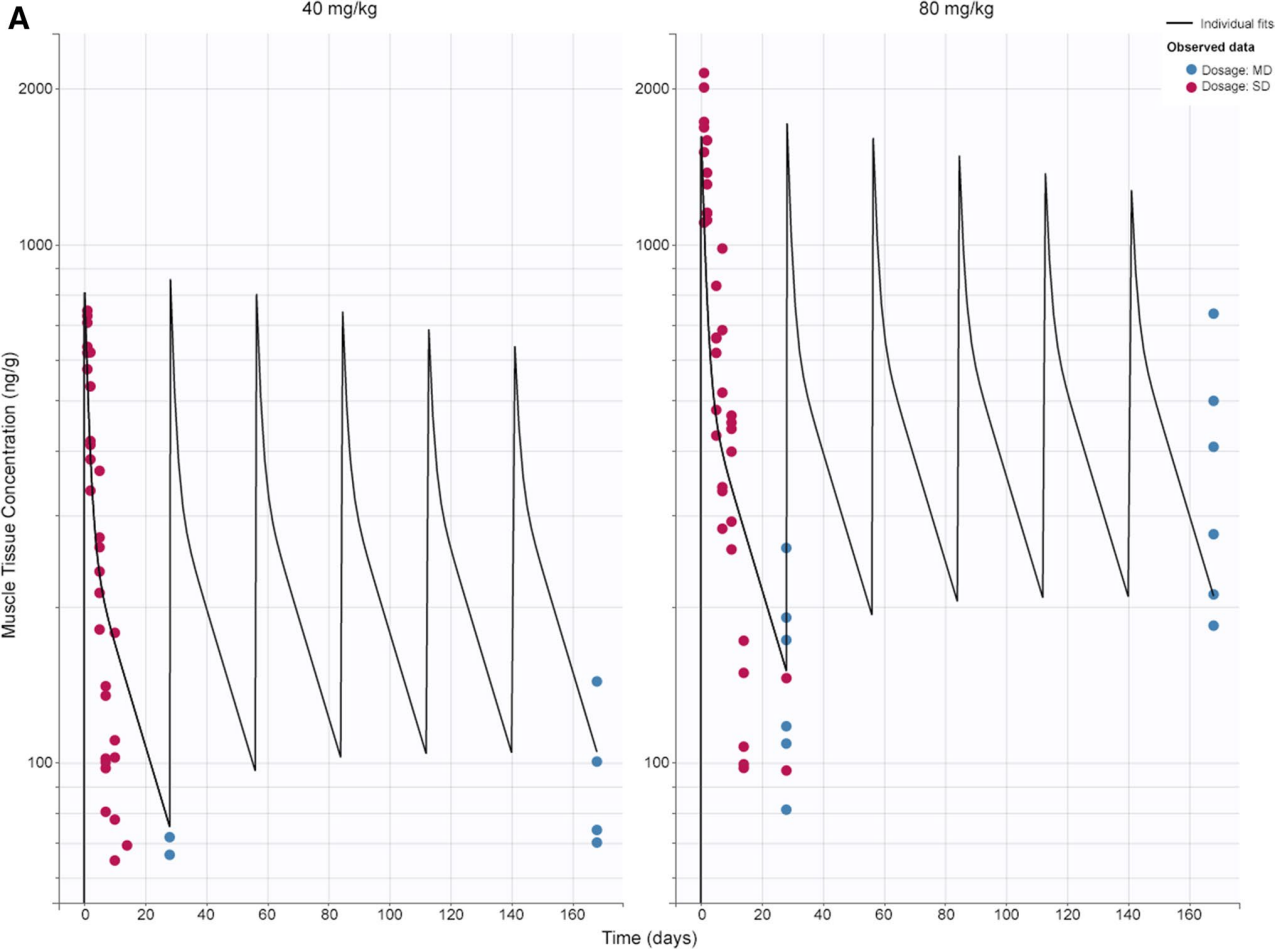

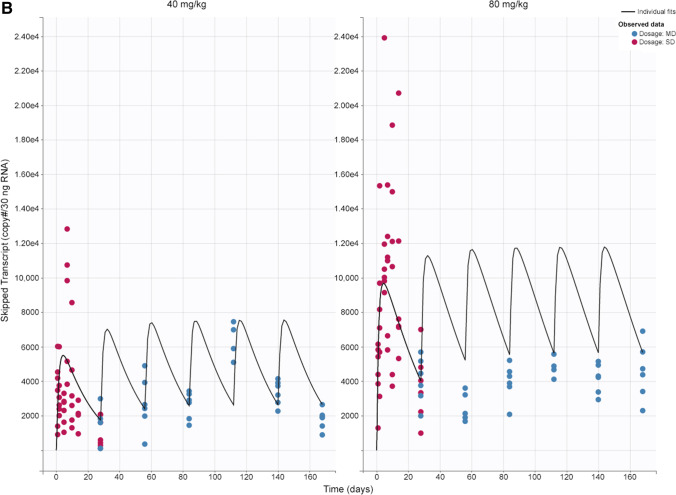

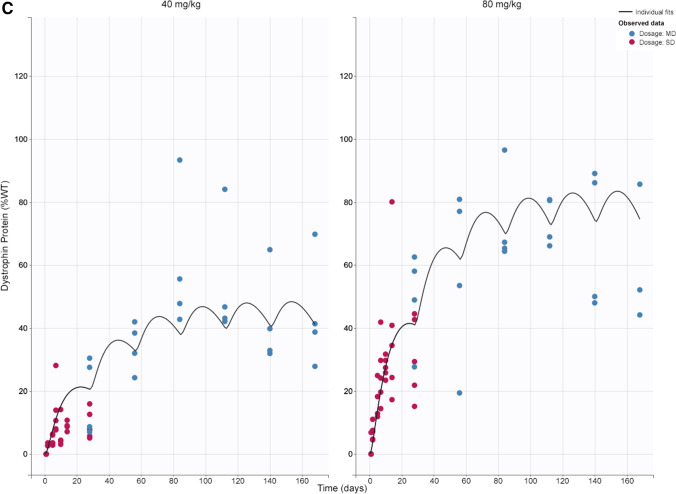
Tissue PK/PD model fitting after single and multiple doses at 40 and 80 mg/kg. Muscle tissue concentration (**a**); skipped transcript (copy numbers per 30 ng total RNA) (**b**); and dystrophin in percent of WT mice (**c**) over time. Lines represent model prediction; dots represent observed data (pink: single dose; blue: multiple dose) at each timepoint (n = 6). *MD* multiple dose, *SD* single dose, *WT* wild-type

**Fig. 5:**
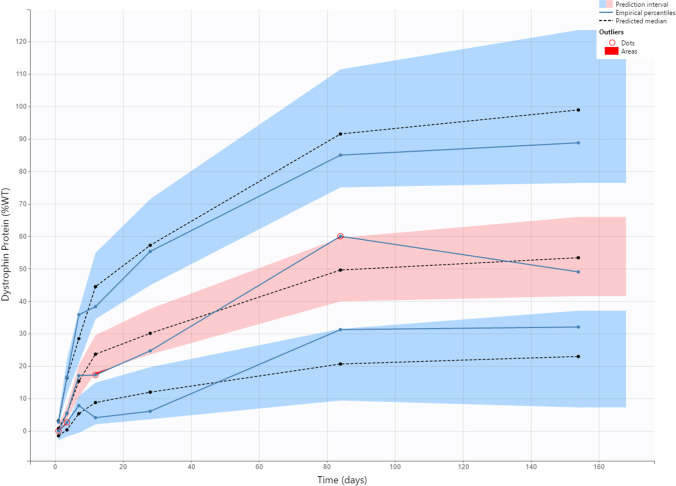
Muscle PK/PD model validation by VPC of dystrophin. The blue and pink shaded areas represent 90% predictive interval around 10th (bottom blue area), 50th (middle orange area) and 90th (top blue area) percentiles. The blue lines (and dots) represent linear connections of empirical percentiles (10th, 50th, and 90th) of observed data. The black dashed lines represent linear connections of percentiles of simulated data between selected time intervals (bins). Red dots and shading represent the outlier dots and areas. *MD* multiple dose, *SD* single dose, *VPC* visual predictive check, *WT* wild-type

A semi-mechanistic PK/PD model was constructed based on data from single- and multiple-dose studies. IDR models were used to characterize PD effects (skipped transcript and dystrophin level), with skipped transcript as an intermediate biomarker. The model described the data reasonably well and accurately estimated parameters, as indicated by low RSE values (Table 2). Concentration vs time plots show good agreement between model prediction and observed data for tissue concentration, skipped mRNA transcript, and dystrophin levels (Fig. 4). Additionally, plots of predicted versus observed show good alignment around the line of identity (Fig. 1S). Other diagnostic plots, including individual weighted residuals versus time and individual weighted residuals versus observed data, confirm good performance of the model (Fig. 2S). Model validation through analysis of VPC plots shows that the observed data generally align with the simulated data (Figs. 5 and 3S). The VPC plots show some outlier areas, but these are consistent with the inherent variability associated with terminal sampling at each timepoint. Some variability may be attributed to the current datasets being from 2 different studies. Use of tissue peripheral compartment concentration (C_1_) instead of central compartment (C_t_) as the PD driver was proven appropriate as we see good profile alignment of C_1_ and skmRNA (Fig. 4S). The plasma concentration profile falls BLQ (10 ng/mL) 8 h post-dose. The model estimated rate constant of elimination in the tissue PK model (k) suggests a tissue PPMO half-life of 5 days, relating to observed limited concentration accumulation after multiple dosing. The dispassion rate constant of skipped transcript (k_out,s_) corresponds to a half-life of 5 h, corresponding to no accumulation in this biomarker after multiple dosing. On the other hand, the dissipation rate constant for dystrophin expression (k_out,Dys_) corresponds to a half-life of 16 days, corresponding to observed dystrophin accumulation after multiple dosing (from 18%WT to 44%WT at 40 mg/kg and from 50%WT to 80%WT at 80 mg/kg from first to 6^th^ dose) and delay in reaching steady state levels observed after the 6th dose.

## Discussion

This is the first PK/PD model for a PPMO. This next-generation PPMO technology may offer increased cell penetration, exon skipping, and prolonged duration of dystrophin restoration in patients with DMD with a mutation amenable to exon skipping. Using this semi-mechanistic PK/PD model built based on the mechanism of action of PPMOs, we were able to link RC-1001 dosing to plasma concentrations to muscle tissue distribution, then to exon skipping, and finally to dystrophin expression using single- and repeat-dose PK/PD studies. Furthermore, the developed PK/PD model may help describe the time course of the PK and PD of future PPMOs for DMD and other disease states. Similar models were used to describe the PK and PD of other ASOs, including an apolipoprotein B-reducing ASO [21] and a survivin mRNA-inhibiting ASO (LY2181308) [28]. With inhibitory mechanisms of action, IDR models describing inhibition of synthesis (IDR model 1) or stimulation of dissipation (IDR model 4) were used to describe their PD data. In contrast, PPMOs lead to synthesis of skipped transcripts, which translate to a functional protein. Therefore, IDR models describing stimulation of synthesis (IDR model 3) were used to describe skipped transcript and dystrophin.

After IV administration of RC-1001, the estimated plasma half-life was 0.32–0.74 h, which is typical for arginine-rich CPP-conjugated PMOs [29]. The short half-life of RC-1001 is due to quick renal excretion [30], rapid drug distribution in different tissue organs, and abundant peptidase enzymatic activity in plasma that cleave the CPP. Additionally, absorption, distribution, metabolism and excretion studies show that more than 50% of administered PPMO molecules are excreted in urine within 24 h of IV dosing (unpublished data).

RC-1001 showed different dose-proportionality behavior in plasma based on either total (AUC) or maximum (C_max_) exposure. RC-1001 shows linear plasma PK up to 80 mg/kg based on C_max_, but linear (dose-proportionality) plasma PK up to 40 mg/kg based on AUC. The AUC was slightly more than dose-proportional at 80 mg/kg, suggesting that a possible elimination process is being saturated. PPMO molecules are metabolized by proteases that remove the peptide [29]. The linear 2-compartment plasma PK model showed superiority over nonlinear PK despite the nonlinearity observation. It is possible that the tested dose range did not saturate the nonlinear elimination process, thereby preventing accurate estimation of nonlinear parameters. The developed 2-compartment PK model characterized bi-exponential decline in tissue concentration data well, suggesting a barrier to distribution of PPMO to a tissue subcompartment; possibly the nucleus, where the molecule exerts its PD effect by binding to pre-mRNA for exon skipping. The IDR models also described both exon skipping (skipped transcripts) and dystrophin levels. The model parameters were well estimated by the current models as shown by low RSE values.

The developed IDR model follows the mechanism of action of exon skipping PPMOs; it characterizes the synthesis/production of skipped transcript after molecules complementarily bind to a dystrophin pre-mRNA sequence to alter splicing of exon 23, which translates into truncated dystrophin.

The estimated parameters reflect quick synthesis of transcript as shown by a high k_in,S_ value. This rapid synthesis of skipped transcript was observed in other studies where skipping was measurable 2 h post-injection (unpublished data). The estimated degradation rate constant of skipped transcript reflects at a half-life of 5 h, which is close to the measured 16 h half-life of human full dystrophin mRNA in fetal human myotubes [31]. The difference between estimated and literature half-life values may be explained by some discrepancies between the current study and the literature: for example, differences in metrics (PPMO-mediated exon 23 skipped transcript vs full-length transcript); study setup (*in vitro* vs *in vivo*); and/or species (mouse model vs human transcript).

The skipped transcript translocates to the cytoplasm, where it is translated to a truncated, in-frame dystrophin. Dystrophin, a structural protein that links the intracellular cytoskeleton to transmembrane components of the dystrophin glycoprotein complex, is an important biomarker in DMD [32, 33]. This model described observed dystrophin and accumulation of dystrophin in a monthly dosing regimen. Unlike skipped transcript production, dystrophin synthesis is a slow process, as reflected by low k_in,Dys_ values. This was also reflected in other experiments, in which dystrophin is not detectable until 2 days post-injection [19]. The estimated rate constant of loss of dystrophin led to an estimated dystrophin half-life of approximately 15 days, which is lower than the > 3-month half-life described in the literature [19]. This may be due to underestimation of half-life based on the short sampling time in the current study, where the longest time was 28 days post-injection. Additionally, VPC plots show agreement between observed and simulated data, further supporting the potential of this model for predicting dystrophin production in *mdx* mice. Restoration of dystrophin can be challenging to quantify in clinical settings, as this assessment can require open biopsy. It is helpful to have a model that can describe the time-course of dystrophin expression utilizing sparse biopsy data. The current model is able to characterize the time-course of this important DMD biomarker and can be used to predict dystrophin levels after different dosing regimens. By applying inter-species scaling methods such as allometric scaling or concomitant translational modeling incorporating preclinical and clinical data, the current model scheme/framework can be scaled to model and describe clinical exon skipping candidate molecules in patients with DMD. Additionally, the model supports that less frequent dosing (once every 4 weeks) may be feasible for PPMOs, despite their short half-life in plasma. In the clinical setting, less frequent dosing has the potential to reduce patient burden for patients with DMD and their caregivers.

## Conclusion

This model characterizes PK/PD profiles of a PPMO and dystrophin accumulation in muscle after single or repeated monthly dosing, and provides a mechanistic explanation for various phases of PK/PD responses observed in *mdx* mice. This first PK/PD model of a PPMO offers a framework for other PPMO molecules and exon skipping ASOs. If applied to other species, it has potential to inform development of future PPMO clinical programs.

## Supplementary Information

Below is the link to the electronic supplementary material.

Fig. 1S: Observed versus prediction plots of tissue concentration (a), skipped transcript (b), and dystrophin in percent of WT mice (c). Line represents unit line and dots represent individual datapoint from each mouse. Abbreviations: MD, multiple dose; SD, single dose; WT, wild-type. Supplementary file1 (PNG 63 kb)

Supplementary file2 (PNG 80 kb)

Supplementary file3 (PNG 60 kb)

Fig. 2S: Plots of residuals for tissue concentration (a), skipped transcript (b), and dystrophin (c). Abbreviations: Ct, predicted muscle tissue concentration; Dys, predicted dystrophin; IWRES, individual weighted residuals; MD, multiple dose; SD, single dose; sk.mRNA, predicted skipped transcript. Supplementary file4 (PNG 69 kb)

Supplementary file5 (PNG 89 kb)

Fig. 3S: Tissue concentration (a) and exon skipping (b) VPC plots. The blue and pink shaded areas represent 90% predictive interval around 10th (lower blue area), 50th (middle orange area), and 90th (upper blue area) percentiles. The blue lines (and dots) represent linear connections of empirical percentiles (10th, 50th, and 90th) of observed data. The black dashed lines represent linear connections of percentiles of simulated data between selected time intervals (bins). Red dots and shading represents the outlier datapoints and areas. Abbreviations: MD, multiple dose; SD, single dose; VPC, visual predictive check. Supplementary file6 (PNG 77 kb)

Supplementary file7 (PNG 77 kb)

Fig. 4S: Profiles of simulated plasma (Cp), tissue central (Ct) and peripheral (C1) compartments concentrations and skipped transcript (skmRNA) after single dose. Plasma concentration falls below limit of quantification (10 ng/mL) after 8 hours, whereas Ct, C1, and skmRNA are still detectable 28 days after dosing. The left y-axis represents tissue central and peripheral compartments concentration (ng/g) and plasma concentration (ng/mL). The right y-axis represents copy numbers of skipped transcripts per 30 ng RNA as measured by ddPCR. The x-axis represents time (day). Supplementary file8 (PNG 292 kb)

Supplementary file9 (PNG 163 kb)

## Abbreviations

AEBSF: 4-(2-Aminoethyl)benzenesulfonyl fluoride hydrochloride
ASO: Antisense oligonucleotide
AUC: Area under the plasma-time curve
BQL: Below the limit of quantification
CI: Confidence interval
CL: Plasma clearance
C_max_: Maximum observed concentration
CPP: Cell-penetrating peptide
CV: Coefficient of variation
ddPCR: Digital droplet polymerase chain reaction
DMD: Duchenne muscular dystrophy
HRP: Horseradish peroxidase
HCI: Hummel’s equivalence criterion interval
IDR: Indirect response
IgG: Immunoglobulin G
IV: Intravenous
NCA: Noncompartmental analysis
PD: Pharmacodynamics
PK: Pharmacokinetics
PMO: Phosphorodiamidate morpholino oligomer
PPMO: Peptide-conjugated phosphorodiamidate morpholino oligomer
RSE: Relative standard error
T_1/2_: Elimination half-life
VPC: Visual predictive check
V_ss_: Volume of distribution
WT: Wild-type
