# Supplementary figures and images for: Pharmacokinetic/Pharmacodynamic Modeling of a Cell-Penetrating Peptide Phosphorodiamidate Morpholino Oligomer in mdx Mice
Source: Pharm Res. 2021 Oct 20;38(10):1731–45. doi: 10.1007/s11095-021-03118-5 (PMC8602220; doi:10.1007/s11095-021-03118-5)

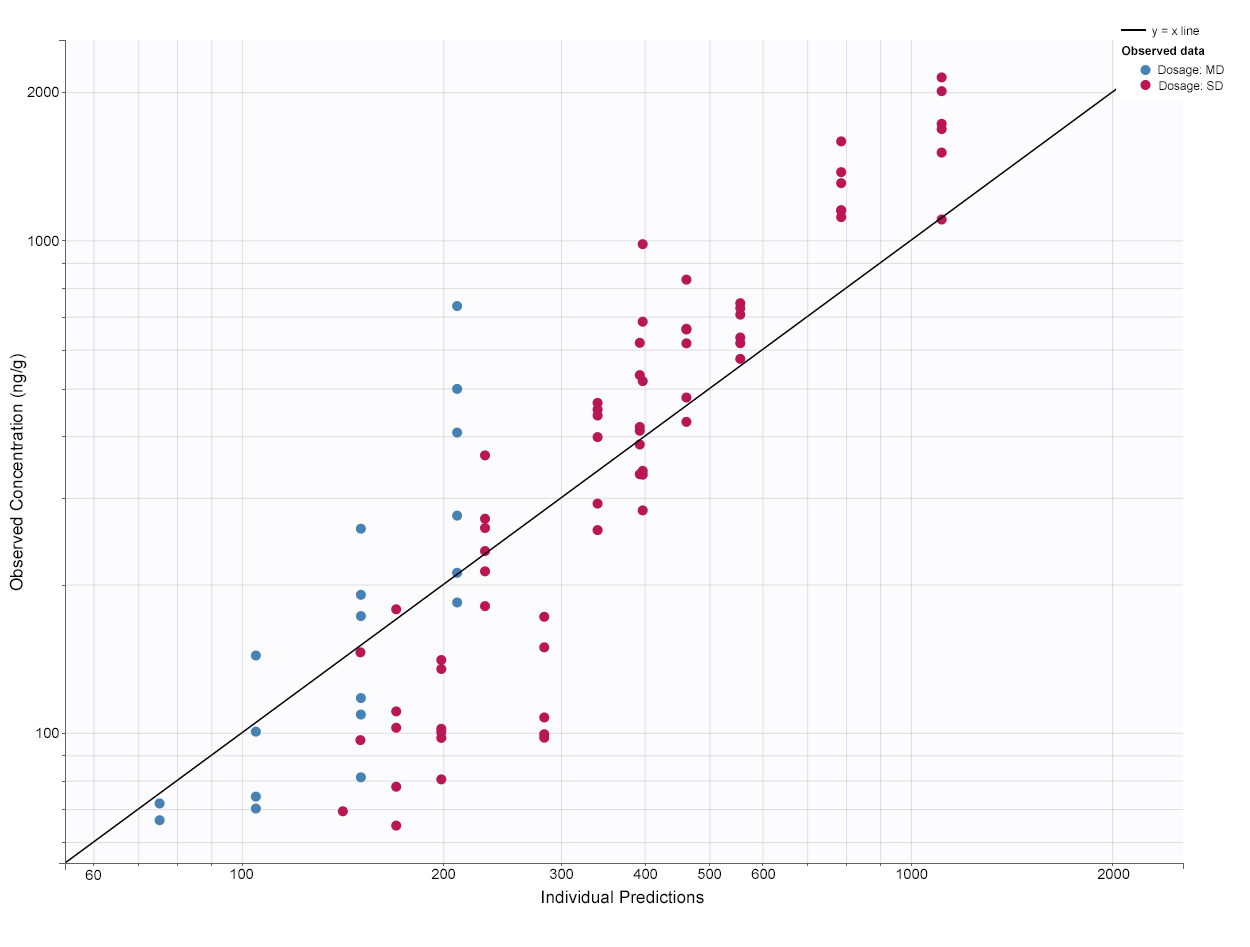

Supplement: Supplementary file 1 — Fig. 1S: Observed versus prediction plots of tissue concentration (a), skipped transcript (b), and dystrophin in percent of WT mice (c). Line represents unit line and dots represent individual datapoint from each mouse. Abbreviations: MD, multiple dose; SD, single dose; WT, wild-type. Supplementary file1 (PNG 63 kb) [file 11095_2021_3118_MOESM1_ESM.png]

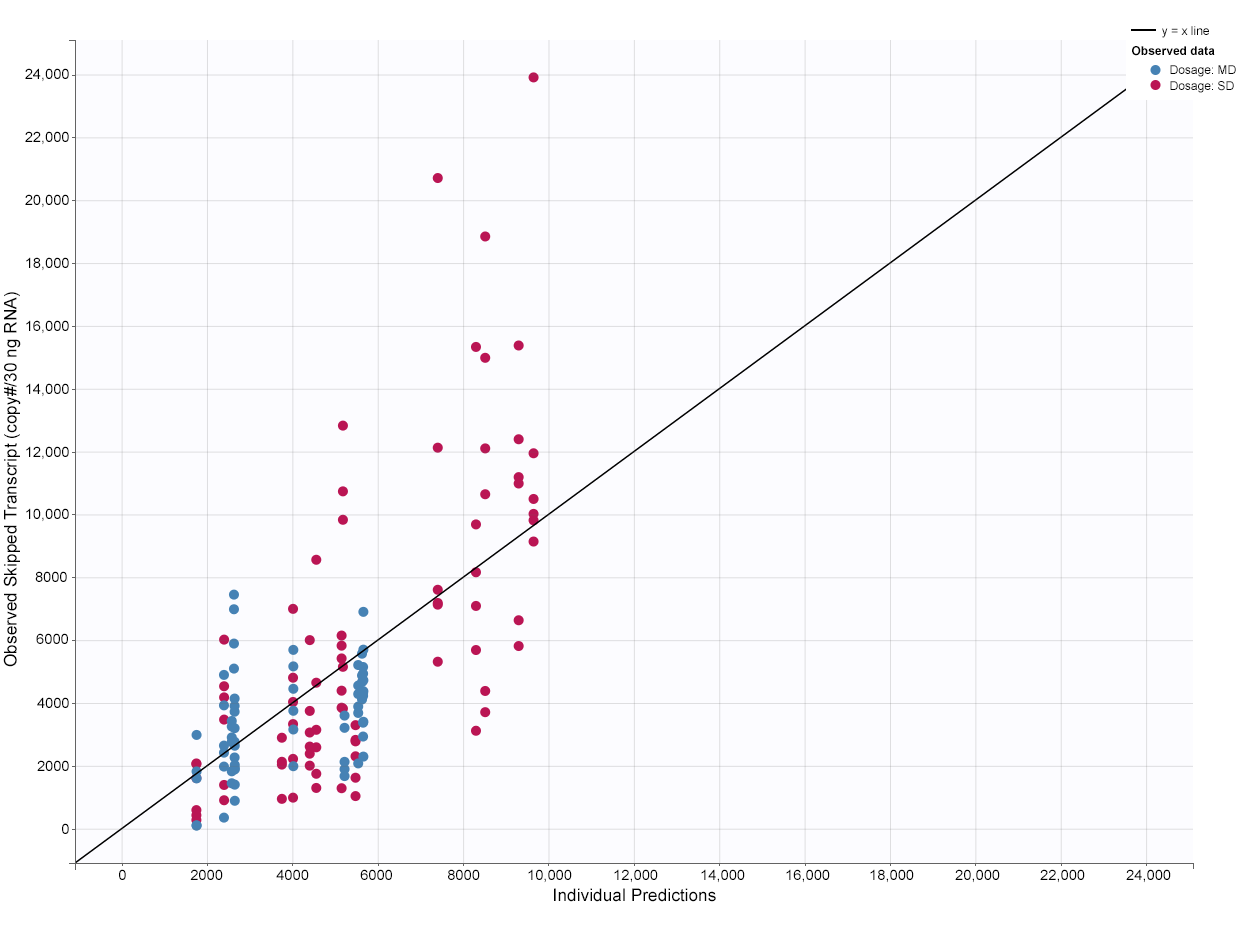

Supplement: Supplementary file 2 — Supplementary file2 (PNG 80 kb) [file 11095_2021_3118_MOESM2_ESM.png]

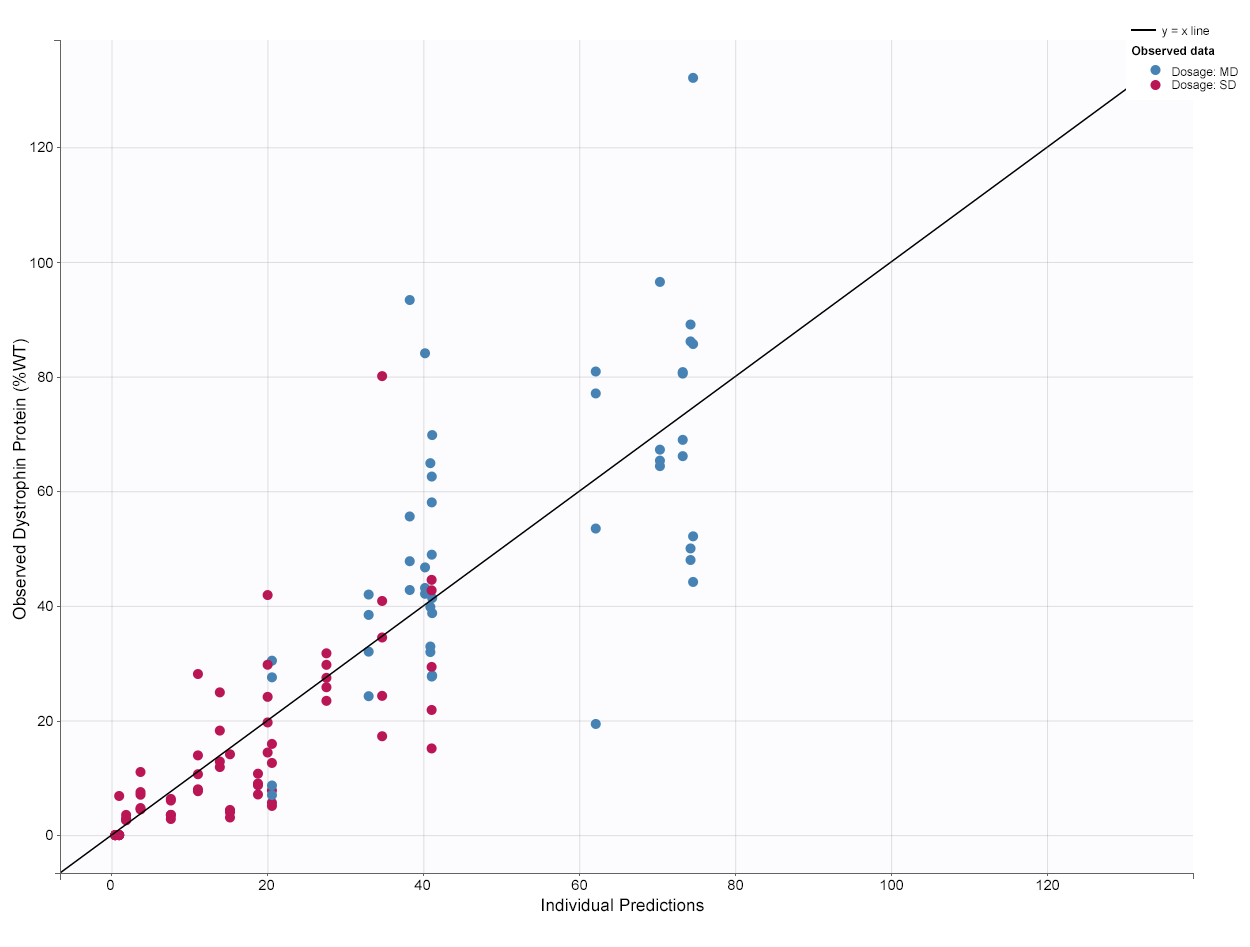

Supplement: Supplementary file 3 — Supplementary file3 (PNG 60 kb) [file 11095_2021_3118_MOESM3_ESM.png]

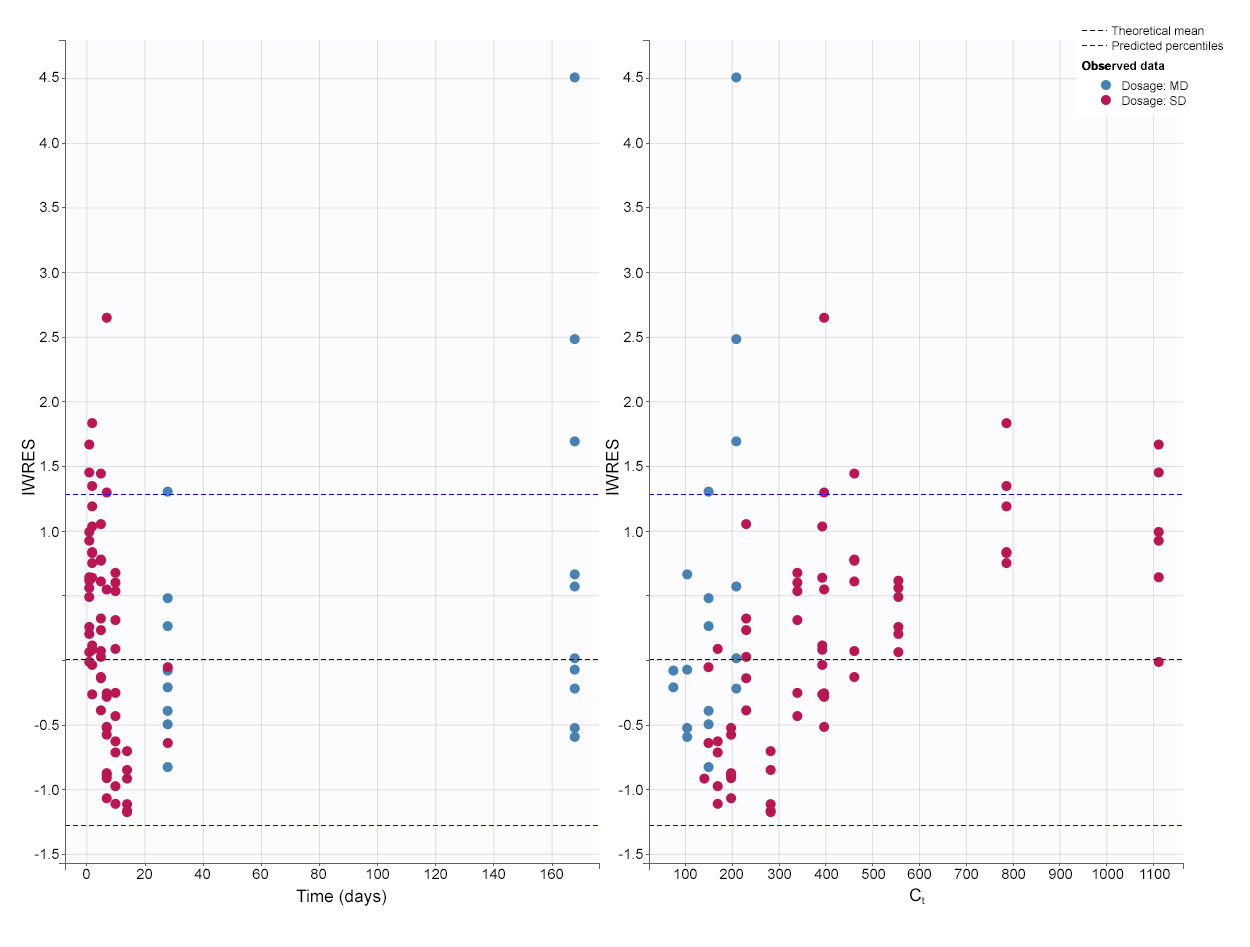

Supplement: Supplementary file 4 — Fig. 2S: Plots of residuals for tissue concentration (a), skipped transcript (b), and dystrophin (c). Abbreviations: Ct, predicted muscle tissue concentration; Dys, predicted dystrophin; IWRES, individual weighted residuals; MD, multiple dose; SD, single dose; sk.mRNA, predicted skipped transcript. Supplementary file4 (PNG 69 kb) [file 11095_2021_3118_MOESM4_ESM.png]

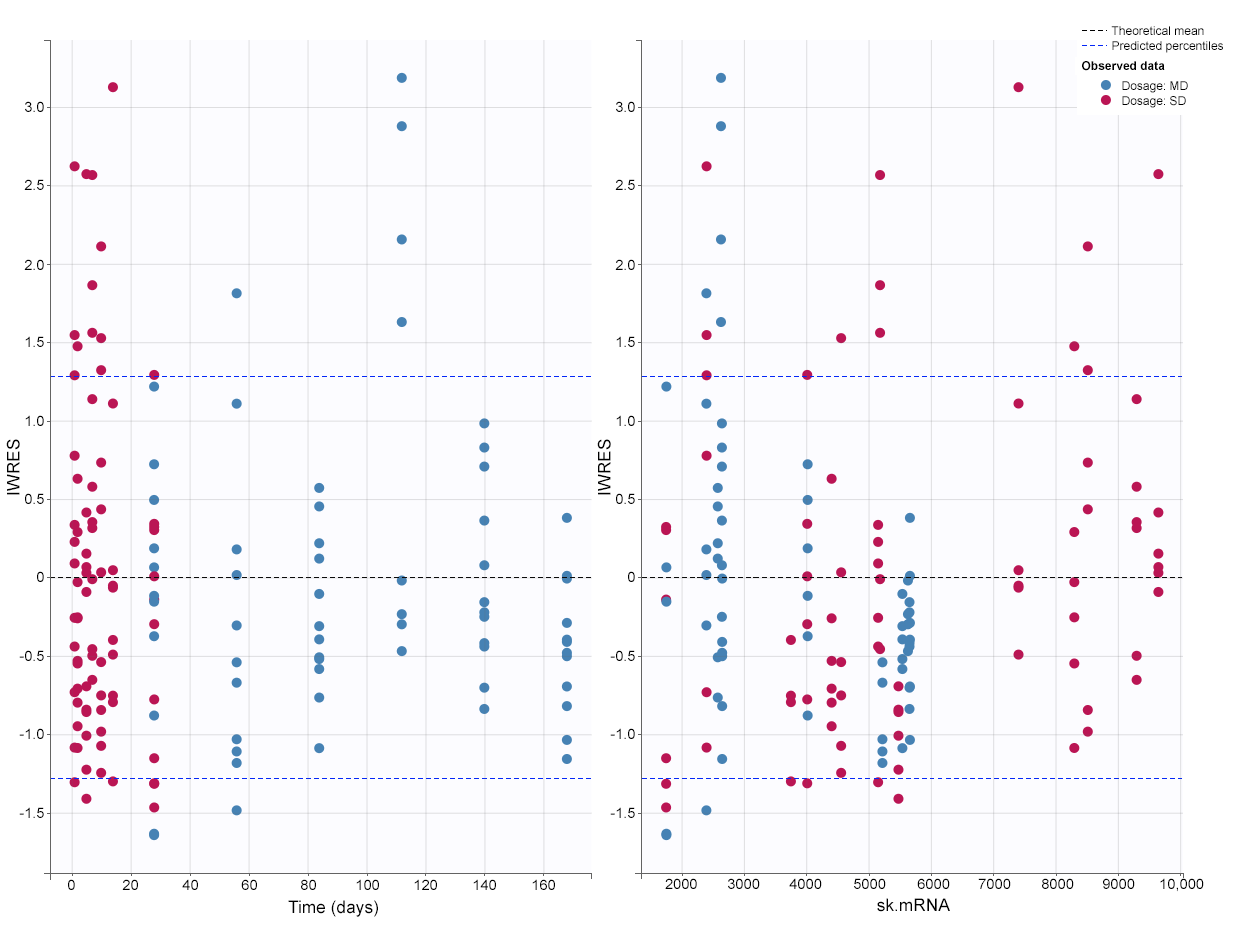

Supplement: Supplementary file 5 — Supplementary file5 (PNG 89 kb) [file 11095_2021_3118_MOESM5_ESM.png]

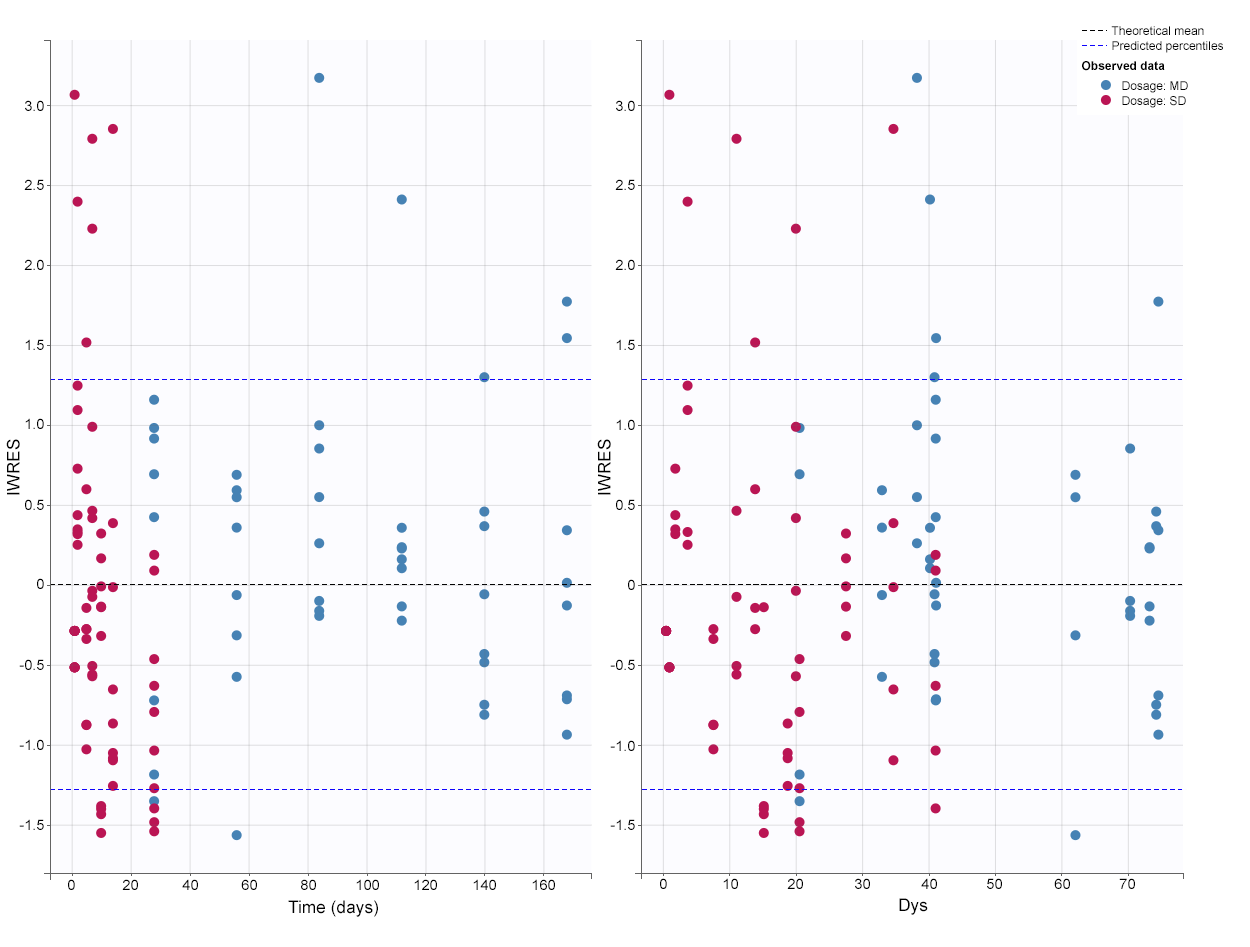

Supplement: Supplementary file 6 — Fig. 3S: Tissue concentration (a) and exon skipping (b) VPC plots. The blue and pink shaded areas represent 90% predictive interval around 10th (lower blue area), 50th (middle orange area), and 90th (upper blue area) percentiles. The blue lines (and dots) represent linear connections of empirical percentiles (10th, 50th, and 90th) of observed data. The black dashed lines represent linear connections of percentiles of simulated data between selected time intervals (bins). Red dots and shading represents the outlier datapoints and areas. Abbreviations: MD, multiple dose; SD, single dose; VPC, visual predictive check. Supplementary file6 (PNG 77 kb) [file 11095_2021_3118_MOESM6_ESM.png]

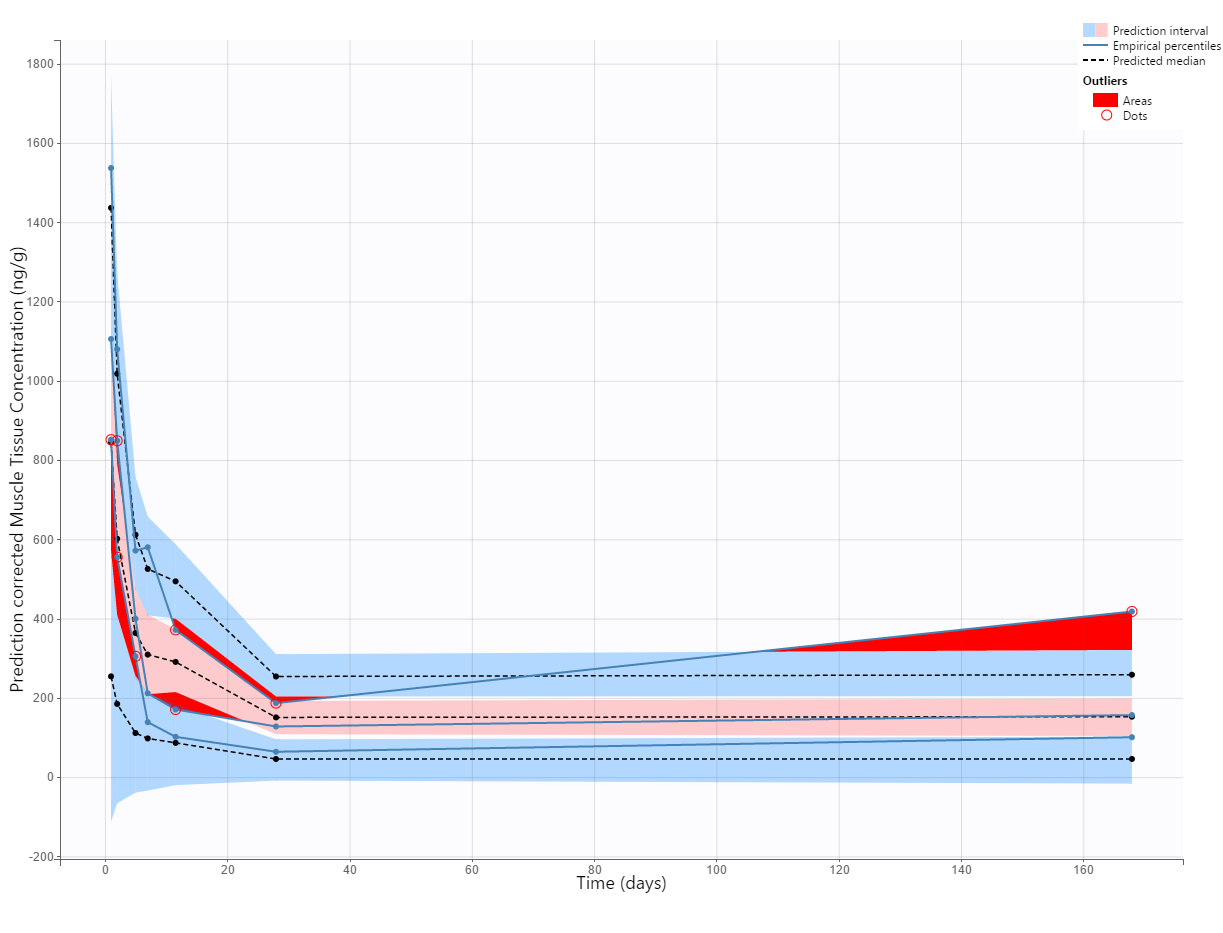

Supplement: Supplementary file 7 — Supplementary file7 (PNG 77 kb) [file 11095_2021_3118_MOESM7_ESM.png]

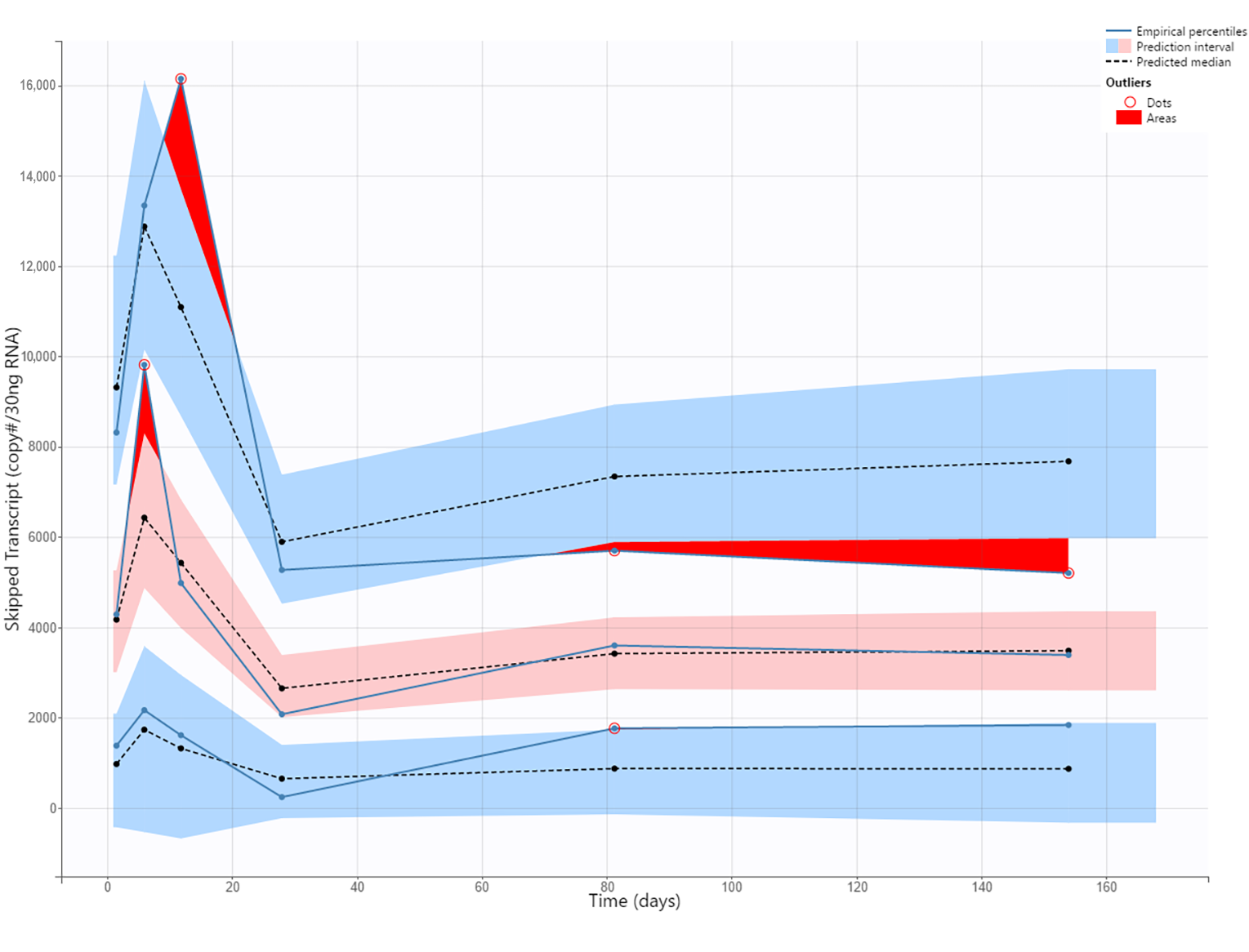

Supplement: Supplementary file 8 — Fig. 4S: Profiles of simulated plasma (Cp), tissue central (Ct) and peripheral (C1) compartments concentrations and skipped transcript (skmRNA) after single dose. Plasma concentration falls below limit of quantification (10 ng/mL) after 8 hours, whereas Ct, C1, and skmRNA are still detectable 28 days after dosing. The left y-axis represents tissue central and peripheral compartments concentration (ng/g) and plasma concentration (ng/mL). The right y-axis represents copy numbers of skipped transcripts per 30 ng RNA as measured by ddPCR. The x-axis represents time (day). Supplementary file8 (PNG 292 kb) [file 11095_2021_3118_MOESM8_ESM.png]

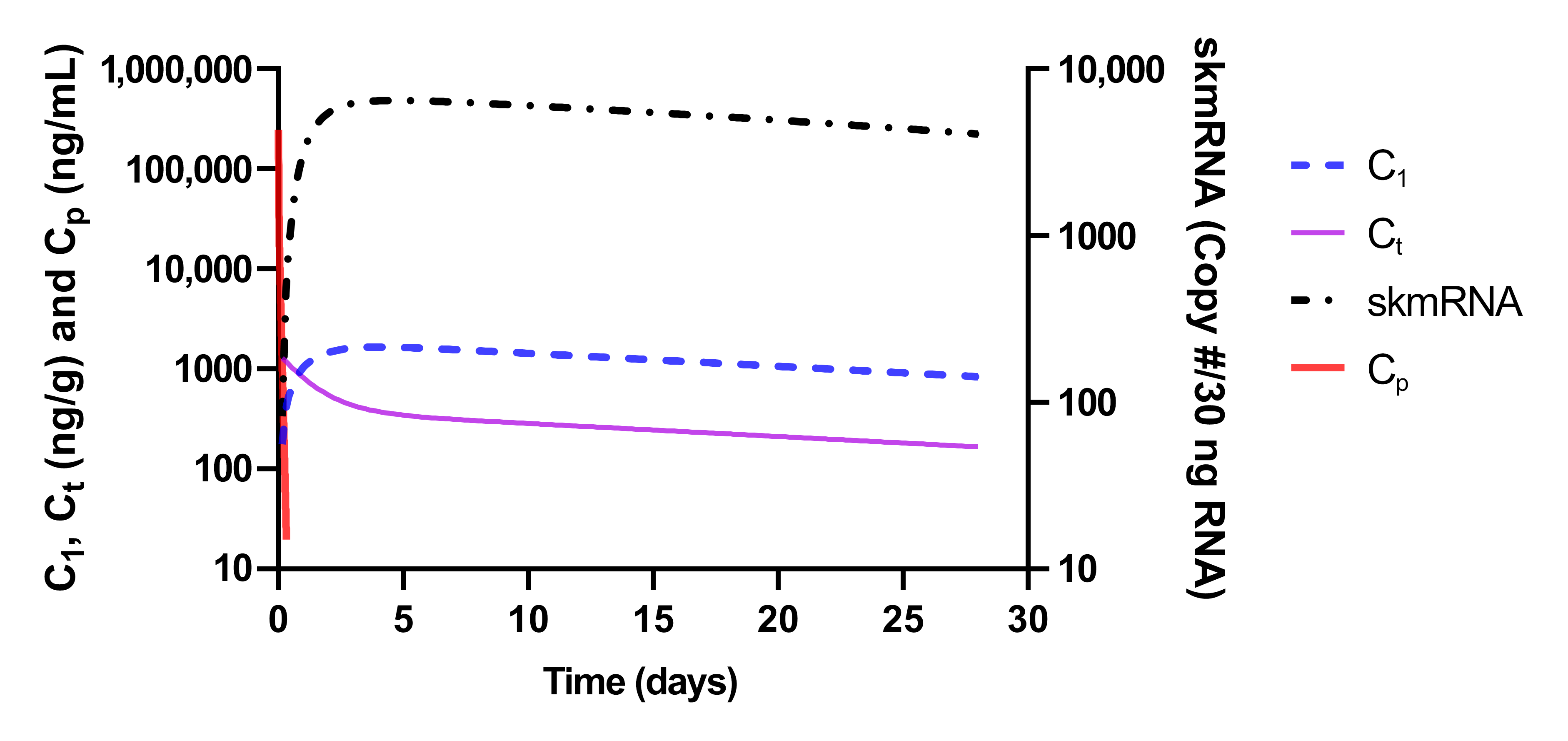

Supplement: Supplementary file 9 — Supplementary file9 (PNG 163 kb) [file 11095_2021_3118_MOESM9_ESM.png]
